# 4-Benz­yloxy-3-(2,4-dichloro­phen­yl)-1-oxaspiro­[4.5]dec-3-en-2-one

**DOI:** 10.1107/S1600536809008356

**Published:** 2009-03-25

**Authors:** Liang-zhong Xu, Qun-qun Su, Jin Huang, Shan-qi Sun

**Affiliations:** a College of Chemistry and Molecular Engineering, Qingdao University of Science and Technology, Qingdao 266042, People’s Republic of China

## Abstract

In the title compound, C_22_H_20_Cl_2_O_3_, the cyclo­hexyl ring adopts a chair conformation. The furanyl ring plane makes dihedral angles of 70.10 (2) and 86.12 (3)° with the 2,4-dichloro­phenyl ring and aromatic ring of the benzyl group, respectively. The crystal structure features weak inter­molecular C—H⋯O and C—H⋯Cl hydrogen bonds.

## Related literature

For similar compounds, see: Bretschneider *et al.* (2003 ▶). For the synthesis, see: Yu *et al.* (1994 ▶); Song *et al.* (2008 ▶). 
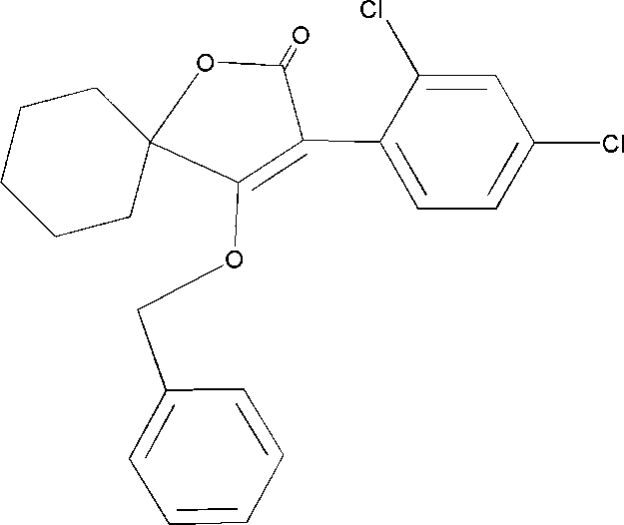


## Experimental

### 

#### Crystal data

C_22_H_20_Cl_2_O_3_
*M* *_r_* = 403.28
Triclinic, 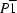

*a* = 7.2624 (15) Å
*b* = 12.117 (2) Å
*c* = 12.679 (3) Å
α = 63.30 (2)°
β = 87.67 (3)°
γ = 73.32 (1)°
*V* = 949.8 (3) Å^3^
*Z* = 2
Mo *K*α radiation
μ = 0.36 mm^−1^
*T* = 113 K
0.18 × 0.16 × 0.10 mm

#### Data collection

Rigaku Saturn diffractometer
Absorption correction: multi-scan (*CrystalClear*; Rigaku, 2005 ▶) *T*_min_ = 0.938, *T*_max_ = 0.965
7095 measured reflections
3328 independent reflections
2376 reflections with *I* > 2σ(*I*)
*R*_int_ = 0.039

#### Refinement

*R*[*F* ^2^ > 2σ(*F* ^2^)] = 0.031
*wR*(*F* ^2^) = 0.078
*S* = 1.03
3328 reflections
244 parameters
H-atom parameters constrained
Δρ_max_ = 0.26 e Å^−3^
Δρ_min_ = −0.22 e Å^−3^

### 

Data collection: *CrystalClear* (Rigaku, 2005 ▶); cell refinement: *CrystalClear*; data reduction: *CrystalClear*; program(s) used to solve structure: *SHELXS97* (Sheldrick, 2008 ▶); program(s) used to refine structure: *SHELXL97* (Sheldrick, 2008 ▶); molecular graphics: *SHELXTL* (Sheldrick, 2008 ▶); software used to prepare material for publication: *SHELXTL*.

## Supplementary Material

Crystal structure: contains datablocks I, global. DOI: 10.1107/S1600536809008356/ng2556sup1.cif

Structure factors: contains datablocks I. DOI: 10.1107/S1600536809008356/ng2556Isup2.hkl

Additional supplementary materials:  crystallographic information; 3D view; checkCIF report

## Figures and Tables

**Table 1:** Hydrogen-bond geometry (Å, °)

*D*—H⋯*A*	*D*—H	H⋯*A*	*D*⋯*A*	*D*—H⋯*A*
C2—H2*A*⋯O2^i^	0.97	2.54	3.477 (2)	162
C2—H2*B*⋯Cl1^ii^	0.97	2.69	3.5051 (19)	142

Symmetry codes: (i) 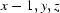; (ii) 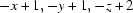.

**Fig. 1.**
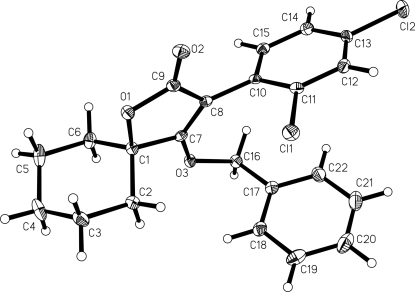
View of the title compound (I), with displacement ellipsoids drawn at the 40% probability level.

## supplementary crystallographic information

### Comment

The title compound (I) was prepared as part of a project in search for new compounds with biological activity (Bretschneider *et al.*, 2003). We report here the crystal structure of (I).

In (I) (Fig. 1), all bond lengths and angles are normal and in a good agreement with those reported previously (Bretschneider *et al.*, 2003). The cyclohexane ring (C1—C6) adopts a chair conformation. The furan ring (O1/C1/C7/C8/C9) plane forms dihedral angles of 71.10 (2)° and 86.12 (3)° with the benzene ring (C10—C15) and the benzyl plane (C16—C22) respectively. In addition to van der Waals forces, the structure is stabilized by weak C—H···O and C—H···Cl hydrogen bonds.

### Experimental

3-(2,4-Dichlorophenyl)-2,4-dioxo-1-oxaspiro[4.5]decane 3.13 g (10.0 mmol), was suspended in a solution of sodium carbonate 0.54 g (5.1 mmol) in 20 ml of water in a flask equipped with stirrer, water separator and reflux condenser. Toluene (40 ml) was added after 0.5 h, the mixture was heated to dehydration to distil the toluene solvent. Then 1-(chloromethyl)benzene 1.39 g (11.0 mmol) and *N*,*N*-dimethylformamide(DMF) solvent (20 ml) were added while maintaining the temperature at 373 K for 4 h. Upon cooling at room temperature water (20 ml) was added. The mixture was extracted with CH_2_Cl_2_ (15 ml) and the organic layer was washed with water and dried over sodium sulfate. The excess CH_2_Cl_2_ was removedon a water vacuum pump to obtain the oily product. Crystallized from methanol to afford the title compound 2.95 g (80% yield) (Yu *et al.*, 1994; Song *et al.*, 2008). Single crystals suitable for X-ray diffraction were obtained by recrystallization from the mixture of acetone and methanol at room temperature.

### Refinement

All C-bound H atoms were placed in calculated positions, with C—H = 0.93–0.97 Å, and included in the final cycles of refinement using a riding model, with *U*_iso_(H) = 1.2*U*_eq_(C) for the aryl and methylene H atoms.

### Crystal data

**Table:**

C_22_H_20_Cl_2_O_3_	*Z* = 2
*M**_r_* = 403.28	*F*(000) = 420
Triclinic, *P*1	*D*_x_ = 1.410 Mg m^−^^3^
Hall symbol: -p 1	Mo *K*α radiation, λ = 0.71073 Å
*a* = 7.2624 (15) Å	Cell parameters from 2983 reflections
*b* = 12.117 (2) Å	θ = 2.0–27.9°
*c* = 12.679 (3) Å	µ = 0.36 mm^−^^1^
α = 63.30 (2)°	*T* = 113 K
β = 87.67 (3)°	Block, colorless
γ = 73.32 (1)°	0.18 × 0.16 × 0.10 mm
*V* = 949.8 (3) Å^3^	

### Data collection

**Table:**

Rigaku Saturn diffractometer	3328 independent reflections
Radiation source: rotating anode	2376 reflections with *I* > 2σ(*I*)
confocal	*R*_int_ = 0.039
ω scans	θ_max_ = 25.0°, θ_min_ = 2.0°
Absorption correction: multi-scan (*CrystalClear*; Rigaku, 2005)	*h* = −8→8
*T*_min_ = 0.938, *T*_max_ = 0.965	*k* = −14→10
7095 measured reflections	*l* = −15→14

### Refinement

**Table:**

Refinement on *F*^2^	Primary atom site location: structure-invariant direct methods
Least-squares matrix: full	Secondary atom site location: difference Fourier map
*R*[*F*^2^ > 2σ(*F*^2^)] = 0.031	Hydrogen site location: inferred from neighbouring sites
*wR*(*F*^2^) = 0.078	H-atom parameters constrained
*S* = 1.03	*w* = 1/[σ^2^(*F*_o_^2^) + (0.03*P*)^2^] where *P* = (*F*_o_^2^ + 2*F*_c_^2^)/3
3328 reflections	(Δ/σ)_max_ = 0.001
244 parameters	Δρ_max_ = 0.26 e Å^−^^3^
0 restraints	Δρ_min_ = −0.22 e Å^−^^3^

### Special details

**Table:**

Geometry. All e.s.d.'s (except the e.s.d. in the dihedral angle between two l.s. planes) are estimated using the full covariance matrix. The cell e.s.d.'s are taken into account individually in the estimation of e.s.d.'s in distances, angles and torsion angles; correlations between e.s.d.'s in cell parameters are only used when they are defined by crystal symmetry. An approximate (isotropic) treatment of cell e.s.d.'s is used for estimating e.s.d.'s involving l.s. planes.
Refinement. Refinement of *F*^2^ against ALL reflections. The weighted *R*-factor *wR* and goodness of fit *S* are based on *F*^2^, conventional *R*-factors *R* are based on *F*, with *F* set to zero for negative *F*^2^. The threshold expression of *F*^2^ > σ(*F*^2^) is used only for calculating *R*-factors(gt) *etc*. and is not relevant to the choice of reflections for refinement. *R*-factors based on *F*^2^ are statistically about twice as large as those based on *F*, and *R*- factors based on ALL data will be even larger.

### Fractional atomic coordinates and isotropic or equivalent isotropic displacement parameters (Å^2^)

**Table:**

	*x*	*y*	*z*	*U*_iso_*/*U*_eq_	
Cl1	0.74501 (6)	0.33929 (4)	1.10256 (4)	0.02229 (14)	
Cl2	1.16128 (7)	−0.12117 (5)	1.44683 (4)	0.02817 (15)	
O1	0.65050 (16)	0.34607 (11)	0.77085 (11)	0.0161 (3)	
O2	0.93505 (17)	0.29879 (11)	0.86875 (11)	0.0195 (3)	
O3	0.37612 (16)	0.16179 (11)	0.97360 (11)	0.0159 (3)	
C1	0.4713 (2)	0.31322 (16)	0.79970 (16)	0.0139 (4)	
C2	0.3139 (3)	0.43289 (16)	0.79041 (17)	0.0181 (4)	
H2A	0.1982	0.4093	0.8194	0.022*	
H2B	0.3566	0.4644	0.8402	0.022*	
C3	0.2661 (3)	0.54029 (17)	0.66320 (18)	0.0236 (5)	
H3A	0.3781	0.5699	0.6365	0.028*	
H3B	0.1616	0.6130	0.6600	0.028*	
C4	0.2071 (3)	0.49221 (18)	0.58136 (19)	0.0313 (5)	
H4A	0.0885	0.4698	0.6038	0.038*	
H4B	0.1827	0.5608	0.5003	0.038*	
C5	0.3647 (3)	0.37423 (18)	0.58838 (17)	0.0295 (5)	
H5A	0.4781	0.3995	0.5568	0.035*	
H5B	0.3201	0.3423	0.5396	0.035*	
C6	0.4199 (3)	0.26580 (16)	0.71537 (16)	0.0199 (4)	
H6A	0.3128	0.2307	0.7424	0.024*	
H6B	0.5296	0.1967	0.7168	0.024*	
C7	0.5194 (2)	0.21147 (15)	0.92748 (16)	0.0131 (4)	
C8	0.6971 (2)	0.19522 (15)	0.96996 (16)	0.0135 (4)	
C9	0.7788 (3)	0.28199 (16)	0.87102 (17)	0.0156 (4)	
C10	0.8070 (2)	0.11567 (16)	1.08853 (16)	0.0129 (4)	
C11	0.8439 (2)	0.17346 (16)	1.15576 (16)	0.0149 (4)	
C12	0.9524 (2)	0.10299 (16)	1.26515 (16)	0.0172 (4)	
H12	0.9773	0.1439	1.3075	0.021*	
C13	1.0228 (2)	−0.02970 (17)	1.30966 (16)	0.0174 (4)	
C14	0.9885 (2)	−0.09177 (16)	1.24730 (17)	0.0176 (4)	
H14	1.0361	−0.1814	1.2790	0.021*	
C15	0.8826 (2)	−0.01880 (16)	1.13717 (16)	0.0160 (4)	
H15	0.8612	−0.0603	1.0945	0.019*	
C16	0.3825 (3)	0.08932 (16)	1.10127 (16)	0.0162 (4)	
H16A	0.5050	0.0213	1.1312	0.019*	
H16B	0.2807	0.0483	1.1194	0.019*	
C17	0.3590 (2)	0.17132 (17)	1.16439 (17)	0.0168 (4)	
C18	0.2414 (3)	0.29913 (17)	1.11236 (18)	0.0202 (4)	
H18	0.1803	0.3361	1.0361	0.024*	
C19	0.2147 (3)	0.37185 (19)	1.17361 (19)	0.0278 (5)	
H19	0.1380	0.4579	1.1378	0.033*	
C20	0.3018 (3)	0.3168 (2)	1.2875 (2)	0.0347 (6)	
H20	0.2826	0.3653	1.3289	0.042*	
C21	0.4176 (3)	0.1892 (2)	1.3404 (2)	0.0344 (6)	
H21	0.4759	0.1520	1.4175	0.041*	
C22	0.4468 (3)	0.11721 (19)	1.27877 (18)	0.0248 (5)	
H22	0.5260	0.0318	1.3143	0.030*	

### Atomic displacement parameters (Å^2^)

**Table:**

	*U*^11^	*U*^22^	*U*^33^	*U*^12^	*U*^13^	*U*^23^
Cl1	0.0253 (3)	0.0150 (2)	0.0274 (3)	−0.00301 (19)	−0.0009 (2)	−0.0119 (2)
Cl2	0.0312 (3)	0.0302 (3)	0.0146 (3)	−0.0031 (2)	−0.0051 (2)	−0.0062 (2)
O1	0.0157 (7)	0.0161 (6)	0.0147 (7)	−0.0071 (5)	0.0021 (5)	−0.0042 (6)
O2	0.0140 (7)	0.0208 (7)	0.0240 (8)	−0.0086 (6)	0.0042 (6)	−0.0087 (6)
O3	0.0160 (7)	0.0181 (6)	0.0130 (8)	−0.0081 (5)	0.0023 (6)	−0.0049 (6)
C1	0.0143 (9)	0.0136 (9)	0.0138 (10)	−0.0076 (7)	0.0019 (8)	−0.0042 (8)
C2	0.0171 (10)	0.0167 (9)	0.0189 (11)	−0.0040 (8)	0.0009 (8)	−0.0075 (8)
C3	0.0210 (11)	0.0182 (10)	0.0227 (12)	−0.0021 (8)	−0.0022 (9)	−0.0039 (9)
C4	0.0354 (13)	0.0295 (12)	0.0186 (12)	−0.0115 (10)	−0.0100 (10)	−0.0001 (10)
C5	0.0476 (14)	0.0311 (11)	0.0138 (12)	−0.0214 (10)	−0.0007 (10)	−0.0077 (9)
C6	0.0280 (11)	0.0184 (10)	0.0169 (11)	−0.0105 (8)	0.0028 (9)	−0.0090 (9)
C7	0.0147 (9)	0.0117 (9)	0.0163 (11)	−0.0054 (7)	0.0050 (8)	−0.0087 (8)
C8	0.0141 (9)	0.0114 (9)	0.0160 (11)	−0.0030 (7)	0.0024 (8)	−0.0078 (8)
C9	0.0159 (10)	0.0128 (9)	0.0179 (11)	−0.0018 (8)	0.0011 (8)	−0.0082 (8)
C10	0.0080 (9)	0.0159 (9)	0.0154 (11)	−0.0037 (7)	0.0028 (7)	−0.0076 (8)
C11	0.0127 (9)	0.0136 (9)	0.0177 (11)	−0.0039 (7)	0.0027 (8)	−0.0068 (8)
C12	0.0178 (10)	0.0214 (10)	0.0162 (11)	−0.0090 (8)	0.0040 (8)	−0.0102 (9)
C13	0.0136 (9)	0.0225 (10)	0.0126 (11)	−0.0043 (8)	0.0012 (8)	−0.0058 (8)
C14	0.0183 (10)	0.0147 (9)	0.0168 (11)	−0.0041 (8)	0.0034 (8)	−0.0053 (8)
C15	0.0144 (10)	0.0182 (9)	0.0187 (11)	−0.0074 (8)	0.0047 (8)	−0.0101 (8)
C16	0.0166 (10)	0.0157 (9)	0.0140 (11)	−0.0091 (8)	0.0046 (8)	−0.0025 (8)
C17	0.0132 (9)	0.0230 (10)	0.0162 (11)	−0.0114 (8)	0.0073 (8)	−0.0075 (8)
C18	0.0197 (10)	0.0246 (10)	0.0174 (11)	−0.0094 (8)	0.0071 (8)	−0.0093 (9)
C19	0.0269 (11)	0.0285 (11)	0.0358 (14)	−0.0124 (9)	0.0150 (10)	−0.0199 (10)
C20	0.0326 (13)	0.0555 (15)	0.0384 (15)	−0.0225 (11)	0.0141 (11)	−0.0358 (13)
C21	0.0277 (12)	0.0596 (15)	0.0243 (13)	−0.0180 (11)	0.0051 (10)	−0.0236 (12)
C22	0.0195 (11)	0.0326 (11)	0.0194 (12)	−0.0082 (9)	0.0024 (9)	−0.0091 (10)

### Geometric parameters (Å, °)

**Table:**

Cl1—C11	1.7325 (18)	C8—C9	1.464 (3)
Cl2—C13	1.7420 (19)	C8—C10	1.477 (2)
O1—C9	1.371 (2)	C10—C15	1.396 (2)
O1—C1	1.454 (2)	C10—C11	1.397 (2)
O2—C9	1.206 (2)	C11—C12	1.383 (2)
O3—C7	1.335 (2)	C12—C13	1.379 (2)
O3—C16	1.449 (2)	C12—H12	0.9300
C1—C7	1.507 (3)	C13—C14	1.382 (2)
C1—C6	1.523 (2)	C14—C15	1.382 (2)
C1—C2	1.526 (2)	C14—H14	0.9300
C2—C3	1.522 (3)	C15—H15	0.9300
C2—H2A	0.9700	C16—C17	1.504 (2)
C2—H2B	0.9700	C16—H16A	0.9700
C3—C4	1.524 (3)	C16—H16B	0.9700
C3—H3A	0.9700	C17—C22	1.386 (3)
C3—H3B	0.9700	C17—C18	1.390 (2)
C4—C5	1.522 (3)	C18—C19	1.386 (3)
C4—H4A	0.9700	C18—H18	0.9300
C4—H4B	0.9700	C19—C20	1.377 (3)
C5—C6	1.526 (3)	C19—H19	0.9300
C5—H5A	0.9700	C20—C21	1.384 (3)
C5—H5B	0.9700	C20—H20	0.9300
C6—H6A	0.9700	C21—C22	1.382 (3)
C6—H6B	0.9700	C21—H21	0.9300
C7—C8	1.345 (2)	C22—H22	0.9300
			
C9—O1—C1	109.44 (14)	O2—C9—C8	129.10 (17)
C7—O3—C16	119.28 (14)	O1—C9—C8	110.02 (15)
O1—C1—C7	102.54 (14)	C15—C10—C11	117.08 (16)
O1—C1—C6	109.19 (15)	C15—C10—C8	122.16 (15)
C7—C1—C6	114.33 (14)	C11—C10—C8	120.74 (14)
O1—C1—C2	108.53 (13)	C12—C11—C10	122.57 (15)
C7—C1—C2	109.95 (15)	C12—C11—Cl1	118.35 (13)
C6—C1—C2	111.78 (14)	C10—C11—Cl1	119.05 (13)
C3—C2—C1	111.71 (16)	C13—C12—C11	118.09 (16)
C3—C2—H2A	109.3	C13—C12—H12	121.0
C1—C2—H2A	109.3	C11—C12—H12	121.0
C3—C2—H2B	109.3	C12—C13—C14	121.61 (16)
C1—C2—H2B	109.3	C12—C13—Cl2	119.22 (14)
H2A—C2—H2B	107.9	C14—C13—Cl2	119.17 (14)
C2—C3—C4	110.71 (16)	C13—C14—C15	119.12 (16)
C2—C3—H3A	109.5	C13—C14—H14	120.4
C4—C3—H3A	109.5	C15—C14—H14	120.4
C2—C3—H3B	109.5	C14—C15—C10	121.52 (16)
C4—C3—H3B	109.5	C14—C15—H15	119.2
H3A—C3—H3B	108.1	C10—C15—H15	119.2
C5—C4—C3	110.90 (16)	O3—C16—C17	113.48 (14)
C5—C4—H4A	109.5	O3—C16—H16A	108.9
C3—C4—H4A	109.5	C17—C16—H16A	108.9
C5—C4—H4B	109.5	O3—C16—H16B	108.9
C3—C4—H4B	109.5	C17—C16—H16B	108.9
H4A—C4—H4B	108.0	H16A—C16—H16B	107.7
C4—C5—C6	112.11 (18)	C22—C17—C18	119.09 (17)
C4—C5—H5A	109.2	C22—C17—C16	119.83 (16)
C6—C5—H5A	109.2	C18—C17—C16	120.98 (16)
C4—C5—H5B	109.2	C19—C18—C17	120.35 (19)
C6—C5—H5B	109.2	C19—C18—H18	119.8
H5A—C5—H5B	107.9	C17—C18—H18	119.8
C1—C6—C5	111.93 (15)	C20—C19—C18	120.03 (18)
C1—C6—H6A	109.2	C20—C19—H19	120.0
C5—C6—H6A	109.2	C18—C19—H19	120.0
C1—C6—H6B	109.2	C19—C20—C21	120.01 (19)
C5—C6—H6B	109.2	C19—C20—H20	120.0
H6A—C6—H6B	107.9	C21—C20—H20	120.0
O3—C7—C8	134.71 (17)	C22—C21—C20	120.0 (2)
O3—C7—C1	113.78 (15)	C22—C21—H21	120.0
C8—C7—C1	111.46 (17)	C20—C21—H21	120.0
C7—C8—C9	106.26 (16)	C21—C22—C17	120.49 (18)
C7—C8—C10	133.14 (17)	C21—C22—H22	119.8
C9—C8—C10	120.56 (15)	C17—C22—H22	119.8
O2—C9—O1	120.86 (17)		
			
C9—O1—C1—C7	−5.26 (15)	C10—C8—C9—O1	−179.53 (13)
C9—O1—C1—C6	−126.89 (14)	C7—C8—C10—C15	71.9 (2)
C9—O1—C1—C2	111.04 (15)	C9—C8—C10—C15	−110.56 (19)
O1—C1—C2—C3	66.38 (18)	C7—C8—C10—C11	−109.8 (2)
C7—C1—C2—C3	177.80 (15)	C9—C8—C10—C11	67.8 (2)
C6—C1—C2—C3	−54.1 (2)	C15—C10—C11—C12	0.9 (3)
C1—C2—C3—C4	56.4 (2)	C8—C10—C11—C12	−177.49 (16)
C2—C3—C4—C5	−56.7 (2)	C15—C10—C11—Cl1	−177.30 (14)
C3—C4—C5—C6	55.2 (2)	C8—C10—C11—Cl1	4.3 (2)
O1—C1—C6—C5	−68.13 (19)	C10—C11—C12—C13	−1.4 (3)
C7—C1—C6—C5	177.68 (15)	Cl1—C11—C12—C13	176.86 (14)
C2—C1—C6—C5	52.0 (2)	C11—C12—C13—C14	0.6 (3)
C4—C5—C6—C1	−52.9 (2)	C11—C12—C13—Cl2	179.62 (13)
C16—O3—C7—C8	13.0 (3)	C12—C13—C14—C15	0.6 (3)
C16—O3—C7—C1	−164.16 (13)	Cl2—C13—C14—C15	−178.43 (14)
O1—C1—C7—O3	−177.66 (12)	C13—C14—C15—C10	−1.0 (3)
C6—C1—C7—O3	−59.62 (19)	C11—C10—C15—C14	0.3 (3)
C2—C1—C7—O3	67.05 (18)	C8—C10—C15—C14	178.71 (16)
O1—C1—C7—C8	4.54 (17)	C7—O3—C16—C17	67.88 (19)
C6—C1—C7—C8	122.59 (17)	O3—C16—C17—C22	−149.82 (16)
C2—C1—C7—C8	−110.74 (16)	O3—C16—C17—C18	33.8 (2)
O3—C7—C8—C9	−179.26 (16)	C22—C17—C18—C19	0.9 (3)
C1—C7—C8—C9	−2.11 (18)	C16—C17—C18—C19	177.25 (18)
O3—C7—C8—C10	−1.4 (3)	C17—C18—C19—C20	−1.3 (3)
C1—C7—C8—C10	175.71 (16)	C18—C19—C20—C21	0.7 (3)
C1—O1—C9—O2	−177.12 (14)	C19—C20—C21—C22	0.3 (3)
C1—O1—C9—C8	4.38 (16)	C20—C21—C22—C17	−0.7 (3)
C7—C8—C9—O2	−179.72 (16)	C18—C17—C22—C21	0.1 (3)
C10—C8—C9—O2	2.1 (3)	C16—C17—C22—C21	−176.28 (18)
C7—C8—C9—O1	−1.38 (18)		

### Hydrogen-bond geometry (Å, °)

**Table:**

*D*—H···*A*	*D*—H	H···*A*	*D*···*A*	*D*—H···*A*
C2—H2A···O2^i^	0.97	2.54	3.477 (2)	162
C2—H2B···Cl1^ii^	0.97	2.69	3.5051 (19)	142

Symmetry codes: (i) *x*−1, *y*, *z*; (ii) −*x*+1, −*y*+1, −*z*+2.
